# Supplementary material for: Lactobacillus gasseri LG-G12 Restores Gut Microbiota and Intestinal Health in Obesity Mice on Ceftriaxone Therapy
Source: Foods. 2023 Mar 3;12(5):1092. doi: 10.3390/foods12051092 (PMC10001121; doi:10.3390/foods12051092)

***Lactobacillus gasseri LG-G12*** **restores gut microbiota and intestinal health in obesity mice on ceftriaxone therapy**

Mariana Dias[1*], Vinícius Duarte[2*], Gabriela Alpino[1], Lúcio Mota[3], Sandra Louzano[1], Lisiane Conceição[1], Hilário Mantovani[4], Solange Pereira[1], Leandro Oliveira[5], Tiago Mendes[6], Davide Porcellato[2], Maria do Carmo Peluzio[1]

**Table S1.** Pairwise comparison (G7 vs G3) of PICRUSt2 predicted pathway abundances.

| G7 vs G3 | | | | |
|---|---|---|---|---|
| **pathway** | **description** | **G7: mean rel. freq. (%)** | **G3: mean rel. freq. (%)** | **p-values (corrected)** |
| ARGORNPROST-PWY | arginine, ornithine and proline interconversion | 0.19 | 0.08 | 0.03 |
| FASYN-ELONG-PWY | fatty acid elongation -- saturated | 0.79 | 0.63 | 0.01 |
| FOLSYN-PWY | superpathway of tetrahydrofolate biosynthesis and salvage | 0.59 | 0.49 | 0.05 |
| HEXITOLDEGSUPER-PWY | superpathway of hexitol degradation (bacteria) | 0.31 | 0.11 | 0.05 |
| LACTOSECAT-PWY | lactose and galactose degradation I | 0.14 | 0.03 | 0.02 |
| P122-PWY | heterolactic fermentation | 0.36 | 0.20 | 0.01 |
| P124-PWY | Bifidobacterium shunt | 0.44 | 0.28 | 0.01 |
| P125-PWY | superpathway of (R,R)-butanediol biosynthesis | 0.06 | 0.00 | 0.04 |
| P461-PWY | hexitol fermentation to lactate, formate, ethanol and acetate | 0.24 | 0.06 | 0.03 |
| PENTOSE-P-PWY | pentose phosphate pathway | 0.51 | 0.27 | 0.03 |
| POLYAMINSYN3-PWY | superpathway of polyamine biosynthesis II | 0.04 | 0.01 | 0.00 |
| PWY-2941 | L-lysine biosynthesis II | 0.50 | 0.16 | 0.00 |
| PWY-3781 | aerobic respiration I (cytochrome c) | 0.66 | 0.11 | 0.01 |
| PWY-5265 | peptidoglycan biosynthesis II (staphylococci) | 0.28 | 0.02 | 0.02 |

| | | | | |
|---|---|---|---|---|
| PWY-5837 | 1,4-dihydroxy-2-naphthoate biosynthesis I | 0.18 | 0.03 | 0.02 |
| PWY-5838 | superpathway of menaquinol-8 biosynthesis I | 0.29 | 0.07 | 0.03 |
| PWY-5840 | superpathway of menaquinol-7 biosynthesis | 0.30 | 0.07 | 0.03 |
| PWY-5861 | superpathway of demethylmenaquinol-8 biosynthesis | 0.26 | 0.06 | 0.02 |
| PWY-5863 | superpathway of phylloquinol biosynthesis | 0.20 | 0.04 | 0.02 |
| PWY-5897 | superpathway of menaquinol-11 biosynthesis | 0.29 | 0.07 | 0.03 |
| PWY-5898 | superpathway of menaquinol-12 biosynthesis | 0.29 | 0.07 | 0.03 |
| PWY-5899 | superpathway of menaquinol-13 biosynthesis | 0.29 | 0.07 | 0.03 |
| PWY-5910 | superpathway of geranylgeranyldiphosphate biosynthesis I (via mevalonate) | 0.21 | 0.04 | 0.00 |
| PWY-6126 | superpathway of adenosine nucleotides de novo biosynthesis II | 0.78 | 0.70 | 0.00 |
| PWY-6147 | 6-hydroxymethyl-dihydropterin diphosphate biosynthesis I | 0.50 | 0.42 | 0.03 |
| PWY-6151 | S-adenosyl-L-methionine cycle I | 0.62 | 0.45 | 0.00 |
| PWY-6470 | peptidoglycan biosynthesis V (β-lactam resistance) | 0.33 | 0.06 | 0.01 |
| PWY-6471 | peptidoglycan biosynthesis IV (Enterococcus faecium) | 0.46 | 0.35 | 0.01 |
| PWY-7200 | superpathway of pyrimidine deoxyribonucleoside salvage | 0.43 | 0.32 | 0.03 |
| PWY-7220 | adenosine deoxyribonucleotides de novo biosynthesis II | 0.72 | 0.56 | 0.01 |
| PWY-7222 | guanosine deoxyribonucleotides de novo biosynthesis II | 0.72 | 0.56 | 0.01 |
| PWY-7234 | inosine-5'-phosphate biosynthesis III | 0.49 | 0.16 | 0.00 |
| PWY-7254 | TCA cycle VII (acetate-producers) | 0.33 | 0.11 | 0.04 |
| PWY-7527 | L-methionine salvage cycle III | 0.02 | 0.00 | 0.01 |
| PWY-7539 | 6-hydroxymethyl-dihydropterin diphosphate biosynthesis III (Chlamydia) | 0.52 | 0.43 | 0.02 |
| PWY-922 | mevalonate pathway I | 0.16 | 0.03 | 0.00 |
| PWY0-166 | superpathway of pyrimidine deoxyribonucleotides de novo biosynthesis (E. coli) | 0.50 | 0.43 | 0.03 |
| PWY0-781 | aspartate superpathway | 0.43 | 0.37 | 0.02 |
| REDCITCYC | TCA cycle VIII (helicobacter) | 0.39 | 0.13 | 0.05 |
| TEICHOICACID-PWY | teichoic acid (poly-glycerol) biosynthesis | 0.16 | 0.05 | 0.04 |
| COBALSYN-PWY | adenosylcobalamin salvage from cobinamide I | 0.31 | 0.46 | 0.02 |
| GLUCUROCAT-PWY | superpathway of β-D-glucuronide and D-glucuronate degradation | 0.17 | 0.23 | 0.04 |

| | | | | |
|---|---|---|---|---|
| GLUTORN-PWY | L-ornithine biosynthesis | 0.54 | 0.73 | 0.05 |
| GLYCOCAT-PWY | glycogen degradation I (bacterial) | 0.62 | 0.82 | 0.05 |
| GLYCOGENSYNTH-PWY | glycogen biosynthesis I (from ADP-D-Glucose) | 0.54 | 0.74 | 0.02 |
| P163-PWY | L-lysine fermentation to acetate and butanoate | 0.01 | 0.04 | 0.00 |
| PWY-181 | photorespiration | 0.14 | 0.37 | 0.04 |
| PWY-5005 | biotin biosynthesis II | 0.06 | 0.15 | 0.01 |
| PWY-5505 | L-glutamate and L-glutamine biosynthesis | 0.33 | 0.56 | 0.04 |
| PWY-5509 | adenosylcobalamin biosynthesis from cobyrinate a,c-diamide I | 0.30 | 0.44 | 0.03 |
| PWY-5676 | acetyl-CoA fermentation to butanoate II | 0.05 | 0.14 | 0.02 |
| PWY-6269 | adenosylcobalamin salvage from cobinamide II | 0.30 | 0.45 | 0.03 |
| PWY-6478 | GDP-D-glycero-α-D-manno-heptose biosynthesis | 0.03 | 0.09 | 0.03 |
| PWY-6891 | thiazole biosynthesis II (Bacillus) | 0.13 | 0.33 | 0.02 |
| PWY-6895 | superpathway of thiamin diphosphate biosynthesis II | 0.28 | 0.40 | 0.00 |
| PWY-7013 | L-1,2-propanediol degradation | 0.01 | 0.05 | 0.02 |
| PWY-7315 | dTDP-N-acetylthomosamine biosynthesis | 0.05 | 0.17 | 0.01 |
| PWY-7328 | superpathway of UDP-glucose-derived O-antigen building blocks biosynthesis | 0.18 | 0.35 | 0.03 |

**Table S2.** Pairwise comparison (G9 vs G5) of PICRUSt2 predicted pathway abundances.

| **G9 vs G5** | | | | |
|---|---|---|---|---|
| **pathway** | **description** | **G9: mean rel. freq. (%)** | **G5: mean rel. freq. (%)** | **p-values (corrected)** |
| COMPLETE-ARO-PWY | superpathway of aromatic amino acid biosynthesis | 0.76 | 0.63 | 0.03 |
| ARO-PWY | chorismate biosynthesis I | 0.74 | 0.61 | 0.01 |
| ARGSYNBSUB-PWY | L-arginine biosynthesis II (acetyl cycle) | 0.67 | 0.49 | 0.05 |
| ARGSYN-PWY | L-arginine biosynthesis I (via L-ornithine) | 0.65 | 0.52 | 0.05 |
| DAPLYSINESYN-PWY | L-lysine biosynthesis I | 0.64 | 0.47 | 0.01 |
| OANTIGEN-PWY | O-antigen building blocks biosynthesis (E. coli) | 0.58 | 0.45 | 0.03 |

| | | | | |
|---|---|---|---|---|
| PWY-5505 | L-glutamate and L-glutamine biosynthesis | 0.50 | 0.34 | 0.02 |
| P124-PWY | Bifidobacterium shunt | 0.29 | 0.13 | 0.02 |
| P122-PWY | heterolactic fermentation | 0.23 | 0.12 | 0.04 |
| PWY-5913 | TCA cycle VI (obligate autotrophs) | 0.34 | 0.48 | 0.03 |
| PWY-6353 | purine nucleotides degradation II (aerobic) | 0.15 | 0.29 | 0.03 |
| GLUCARDEG-PWY | D-glucarate degradation I | 0.01 | 0.04 | 0.01 |
| GALACTARDEG-PWY | D-galactarate degradation I | 0.01 | 0.03 | 0.05 |
| GLUCARGALACTSUPER-PWY | superpathway of D-glucarate and D-galactarate degradation | 0.01 | 0.03 | 0.05 |

**Table S3.** Pairwise comparison (G8 vs G4) of PICRUSt2 predicted pathway abundances.

| **G8 vs G4** | | | | |
|---|---|---|---|---|
| **pathway** | **description** | **G8: mean rel. freq. (%)** | **G4: mean rel. freq. (%)** | **p-values (corrected)** |
| FAO-PWY | fatty acid β-oxidation I | 0.07 | 0.02 | 0.013 |
| PWY-5971 | palmitate biosynthesis II (bacteria and plants) | 0.21 | 0.10 | 0.048 |
| PWY-5989 | stearate biosynthesis II (bacteria and plants) | 0.14 | 0.06 | 0.002 |
| PWYG-321 | mycolate biosynthesis | 0.16 | 0.08 | 0.027 |
| ALL-CHORISMATE-PWY | superpathway of chorismate metabolism | 0.01 | 0.03 | 0.019 |
| PWY-5845 | superpathway of menaquinol-9 biosynthesis | 0.01 | 0.04 | 0.025 |
| PWY-5850 | superpathway of menaquinol-6 biosynthesis I | 0.01 | 0.04 | 0.025 |
| PWY-5896 | superpathway of menaquinol-10 biosynthesis | 0.01 | 0.04 | 0.025 |
| PWY-6125 | superpathway of guanosine nucleotides de novo biosynthesis II | 0.35 | 0.97 | 0.042 |
| PWY-7197 | pyrimidine deoxyribonucleotide phosphorylation | 0.22 | 0.81 | 0.043 |
| PWY-7228 | superpathway of guanosine nucleotides de novo biosynthesis I | 0.32 | 0.98 | 0.044 |
| PWY0-162 | superpathway of pyrimidine ribonucleotides de novo biosynthesis | 0.43 | 1.01 | 0.040 |

**Figure S1.** Filtering and denoising Amplicon Sequence Variants (ASVs) statistics following the DADA2 pipeline.

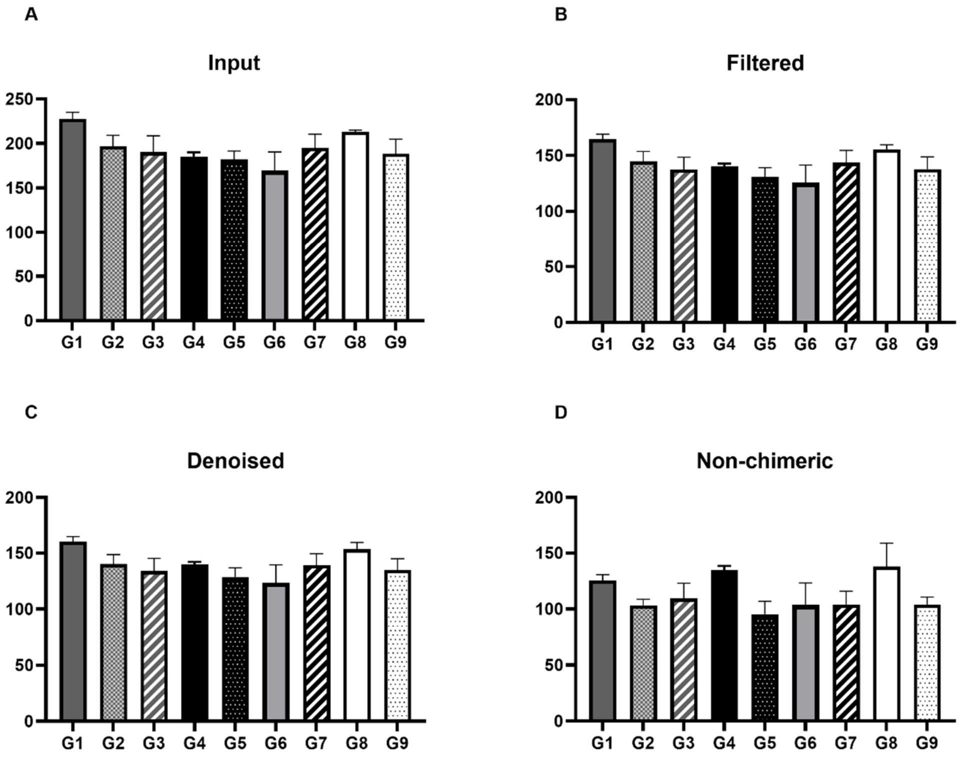

**Figure S2.** Boxplots of the weighted (A) and unweighted (B) UniFrac distances to G1 are shown. Boxes show the 25th and 75th percentiles with the median represented by a horizontal line. Whiskers show the data range, with the exception of outlier, which is indicated by a data point.

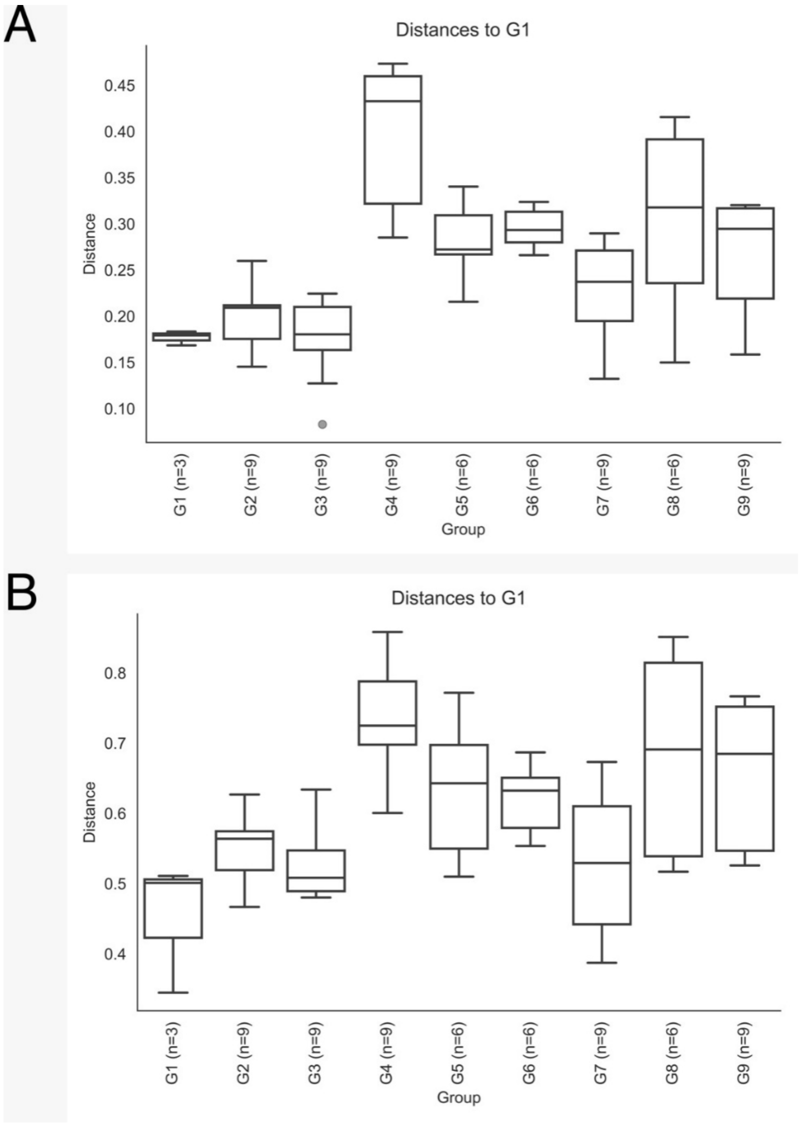

**Figure S3.** Canonical correspondence analysis (CCA) among groups treated with ceftriaxone. CCA was performed on the most abundant OTUs (relative abundance > 0.1%) at the genus level for the groups G1, G2, G4, G6, and G8, with **A)** intestinal permeability parameters (lactulose, mannitol, and lactulose/mannitol ratio) and **B)** the short-chain fatty acids acetate, propionate, butyrate, and total SCFA. Green lines indicate the direction and magnitude of measurable variables associated with community structures.

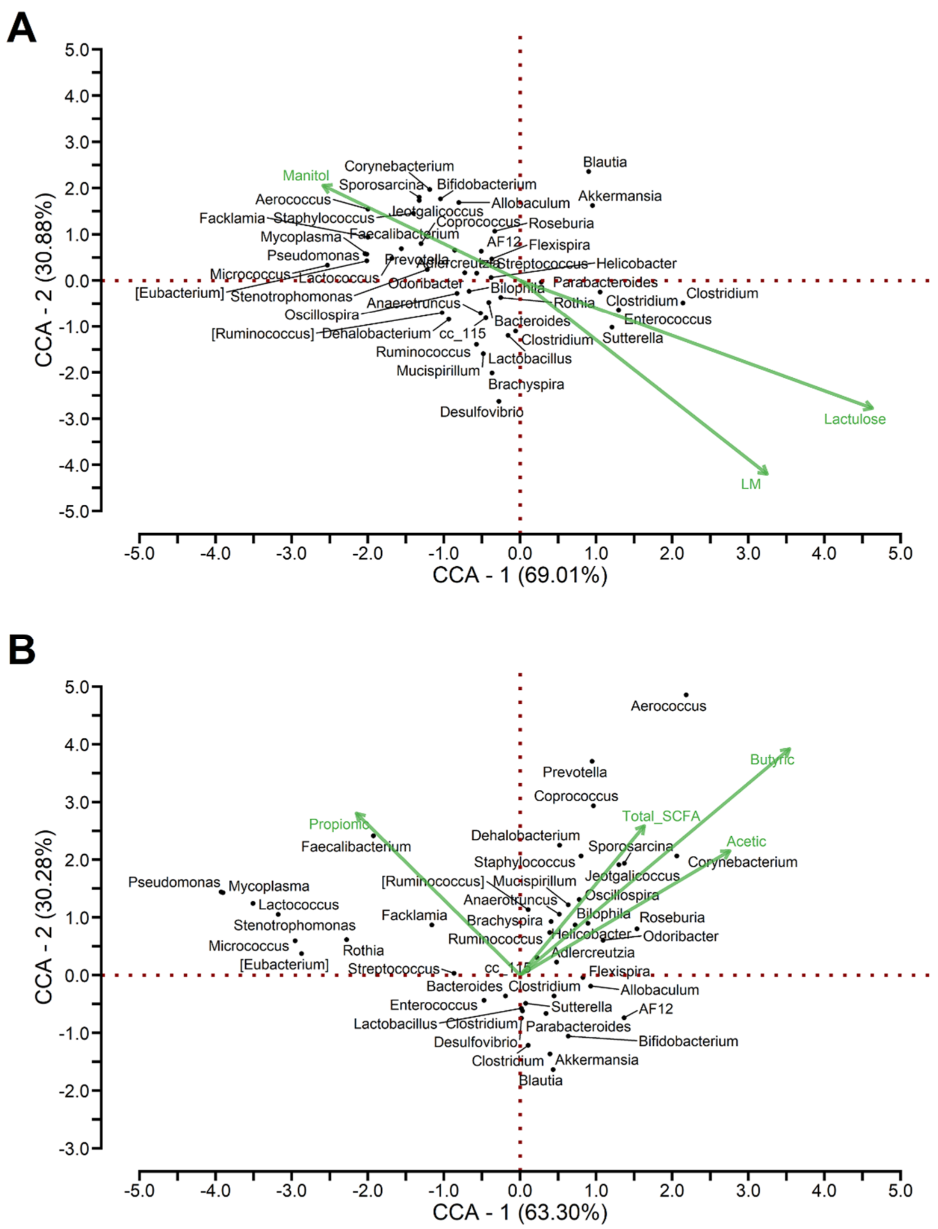

**Figure S4.** Canonical correspondence analysis (CCA) among groups treated with LG-G12 and ceftriaxone. CCA was performed on the most abundant OTUs (relative abundance > 0.1%) at the genus level for the groups G1, G2, G5, G6, and G9, with **A)** intestinal permeability parameters (lactulose, mannitol, and lactulose/mannitol ratio) and **B)** the short-chain fatty acids acetate, propionate, butyrate, and total SCFA. Green lines indicate the direction and magnitude of measurable variables associated with community structures.

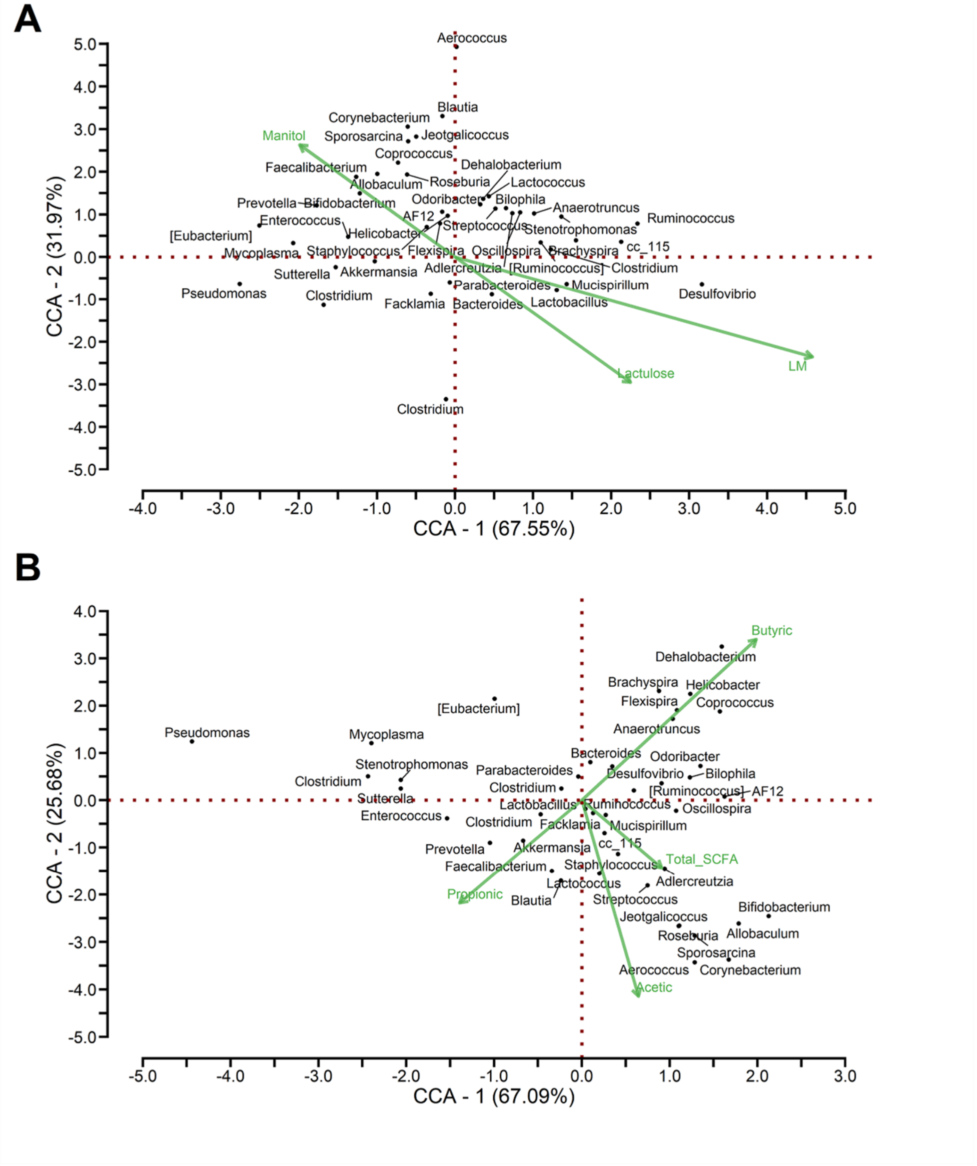

**Figure S5.** Box plot showing the distribution in the proportion of selected MetaCyc pathways between the groups G5 and G9. Boxes indicate the IQR (75th to 25th of the data). The median value is shown as a line within the box and the mean value as a star. Whiskers extend to the most extreme value within 1.5*IQR. Where: a: Bifidobacterium shunt; b: Heterolactic fermentation; c: Superpathway of aromatic amino acids biosynthesis; d: Superpathway of D-glucarate and D-galactarate degradation; e: Purine nucleotides degradation II (aerobic).

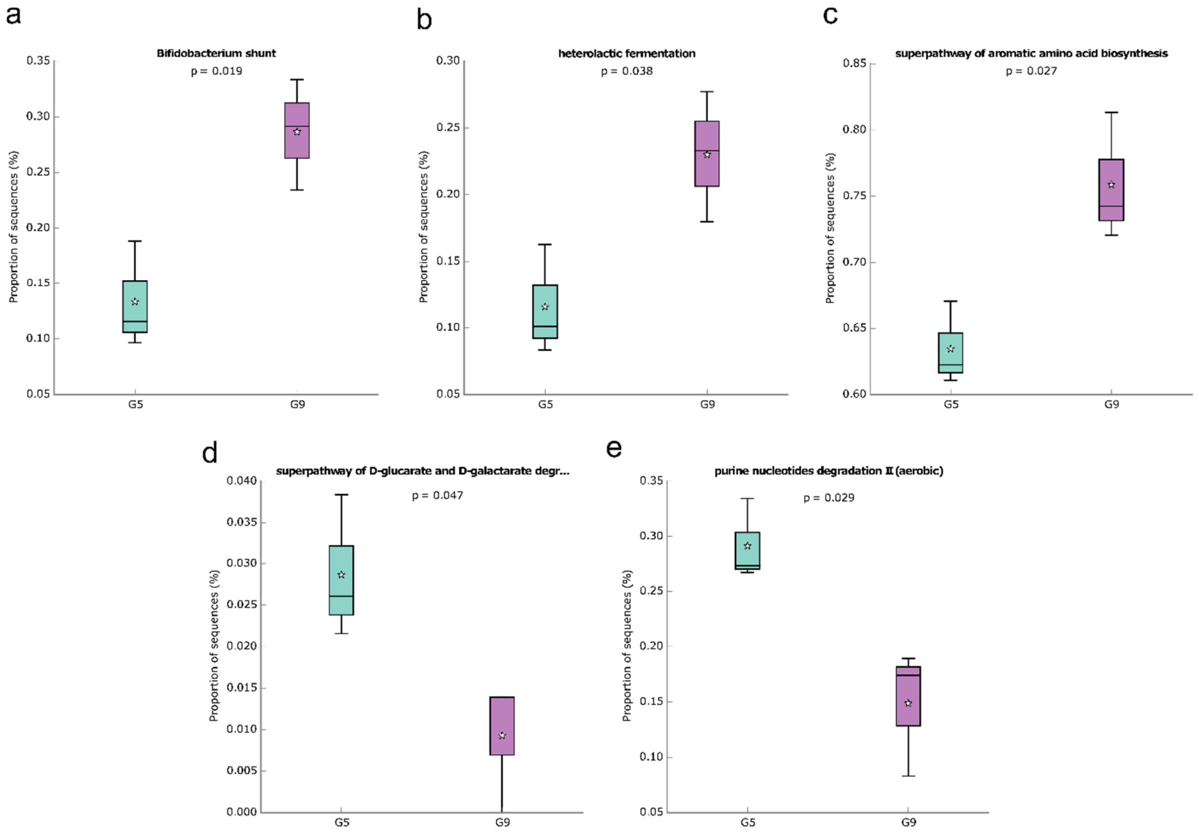

Supplement: Supplementary file 1 [file foods-12-01092-s001.zip › foods-2140124-supplementary.pdf]
